# Periodic Heat Shock Accelerated the Chondrogenic Differentiation of Human Mesenchymal Stem Cells in Pellet Culture

**DOI:** 10.1371/journal.pone.0091561

**Published:** 2014-03-14

**Authors:** Jing Chen, Chenghai Li, Sihong Wang

**Affiliations:** Department of Biomedical Engineering, The City College of the City University of New York, New York, New York, United States of America; University of Pittsburgh, United States of America

## Abstract

Osteoarthritis (OA) is one of diseases that seriously affect elderly people's quality of life. Human mesenchymal stem cells (hMSCs) offer a potential promise for the joint repair in OA patients. However, chondrogenic differentiation from hMSCs *in vitro* takes a long time (∼6 weeks) and differentiated cells are still not as functionally mature as primary isolated chondrocytes, though chemical stimulations and mechanical loading have been intensively studied to enhance the hMSC differentiation. On the other hand, thermal stimulations of hMSC chondrogenesis have not been well explored. In this study, the direct effects of mild heat shock (HS) on the differentiation of hMSCs into chondrocytes in 3D pellet culture were investigated. Periodic HS at 41°C for 1 hr significantly increased sulfated glycosaminoglycan in 3D pellet culture at Day 10 of chondrogenesis. Immunohistochemical and Western Blot analyses revealed an increased expression of collagen type II and aggrecan in heat-shocked pellets than non heat-shocked pellets on Day 17 of chondrogenesis. In addition, HS also upregulated the expression of collagen type I and X as well as heat shock protein 70 on Day 17 and 24 of differentiation. These results demonstrate that HS accelerated the chondrogenic differentiation of hMSCs and induced an early maturation of chondrocytes differentiated from hMSCs. The results of this study will guide the design of future protocols using thermal treatments to facilitate cartilage regeneration with human mesenchymal stem cells.

## Introduction

Articular cartilage is an avascular connective tissue that has limited capacity for self-repair. Partial thickness of articular lesion, which affects only the superficial cartilage, does not heal spontaneously [1]. Injuries that affect deep into the subchondral bone, exhibit a repair process leading to the formation of a fibrocartilage, however is inferior to native articular cartilage [2]. Autologous chondrocyte implantation (ACI), the first cell therapeutic approach introduced in 1994, is currently used in clinical cartilage repair, but it has disadvantages such as limited cell availability, and donor site morbidity [1], [2], [3]. Thus tissue engineering approaches using biodegradable and biocompatible scaffolds with cells and/or growth factors [3], [4], [5] may hold a great promise for functional cartilage regeneration. They may also provide curing solutions to the challenging diseases such as osteoarthritis (OA) which occurs frequently in the aging population.

Due to the low chondrocyte density in articular cartilages and the fact that they tend to dedifferentiate when expanded in 2D culture, there is a shift of cell sources from chondrocytes to adult stem cells for cartilage tissue engineering applications [1], [6]. Human mesenchymal stem cells (hMSCs) isolated from a well-known source, i.e. bone marrow, have multilineage differentiation potential and can be differentiated towards chondrogenic lineage *in vitro* [7], [8]. In addition, they have been tested for cartilage repair potential by mixing with biomaterials like type I collagen in studies in rabbits [9], [10], [11], [12] with promising results and further in some clinical studies [13], [14], [15]. A lot of recent research has focused on effects of growth factors [16], [17], scaffold materials [18], [19], or mechanical loading [20], [21] on MSC differentiation into chondrocytes. However, the extracellular matrix (ECM) content produced by MSCs and mechanical properties of constructs formed by them are inferior to those functional cartilaginous properties of mature primary chondrocyte-seeded constructs [22], [23] even a long time (i.e. 4 to 6 weeks) in vitro differentiation is usually used. It suggests that methods for inducing MSC chondrogenesis have yet to be optimized to enhance the maturation of cells differentiated from MSCs and generate tissue-engineered cartilage that matches native cartilage. The data from earlier studies have shown that the isolated hMSCs within passage 4 retain their differentiation abilities in vitro [24], [25]. Considering significant amount of cells are needed for in vitro tissue regeneration, we chose to use passage 4 hMSCs for this study. Temperature has an important impact on tissue development in general [26], [27]. It may be one of the missing factors in the regulation of the MSC differentiation but has not been well studied.

Hyperthermia has been widely used as a thermotherapy for the musculoskeletal diseases, such as the treatment of articular cartilage with OA [28]. Heat stimulation was known to relieve the pain in OA patients [29], [30], however, the effect has not been sufficiently investigated in clinical studies and the mechanism remains unknown. The direct effect of heat on metabolism and repair of the articular cartilage, the mainly affected tissue of OA, is also unknown. Furthermore, a few studies have shown that heat shock protein (HSP) 70 has a protective effect on the cartilage as it inhibits apoptosis of chondrocytes as well as increases the cartilage metabolism [31]–[37], and thus induction of HSP70 by hyperthermia may help slow down the progression of OA and further prevent cartilage degeneration. Besides, in one study, proteoglycan metabolism in chondrocyte was found to be increased using heat at 41°C [38]. In addition, effect of hyperthermia on the articular cartilage *in vivo* has been studied in a rabbit model [39]. Microwave applied on rabbit knee joint has increased the joint temperature to 40°C and also increased the proteoglycan and type II collagen expression in the articular cartilage [39]. The same study also showed that HSP70 was upregulated in the chondrocytes and confirmed to be partially responsible for the increase in the matrix production [39]. However, no studies have been conducted to investigate thermal effects on bone marrow stem cell differentiation into chondrocytes.

Recently we have shown in an *in vitro* study, periodic heat shock at 41°C enhanced osteogenic differentiation of hMSCs analyzed by alkaline phosphatase activities and calcium deposition in 2D culture and 3D peptide hydrogel culture [40]. On the basis of this finding and the fact that heat treatment of knee joint usually involves both cartilage and bone in OA patients, we hypothesize that the effect of heat on bone and cartilage may be closely correlated with each other. Therefore, the aim of this work was to further investigate direct heat shock effects on hMSC chondrogenesis in 3D pellet culture. The synthesis of sulfated glycosaminoglycans (sGAGs) was assessed biochemically. Immunohistochemical analyses were used to determine the type of ECM produced including collagen type II and aggrecan (the markers for hyaline cartilage), collagen type I (a marker for fibrocartilage), collagen type X (a marker for hypertrophic cartilage), and the expression of heat shock protein (HSP) 70 induced with heat. Results of this study would help elucidate the mechanism of heat stimulation on articular cartilage regeneration *in vivo* and guide the design of a thermotherapy protocol potentially for OA treatments.

## Materials and Methods

All reagents and chemicals without manufacture labels were purchased from Sigma-Aldrich (St Louis, MO).

### hMSC Isolation and Characterization

Human MSCs were isolated from bone marrow (BM) (AllCells LLC, Berkeley, CA) as described previously [40]. Briefly, the BM was mixed with RosetteSep MSC enrichment cocktail (StemCell Technologies, Vancouver, Canada) and incubated for 20 minutes at room temperature. Afterwards, the sample was layered on top of the Ficoll-Paque (StemCell Technologies) density gradient solution and centrifuged for 25 minutes at 300×g. Enriched mononuclear cells were then removed and cultured as passage 0 in tissue culture flasks with hMSC growth medium consisting of Dulbecco's modified Eagle's medium (DMEM)-low glucose, with 10% fetal bovine serum (FBS) (Atlanta Biologicals, Lawrenceville, GA), and 1% penicillin-streptomycin (Invitrogen, Carlsbad, CA). The adherent cells were further expanded at 5000 cells/cm^2^ until passage 4. Prior to the following study, hMSCs were characterized using surface markers by flow cytometry as previously described [40]. Briefly, the cells were stained with antibodies against human CD45, CD44, CD147, CD29, CD34, or CD146 (BD Biosciences, San Jose, CA) and analyzed on a FACSCalibur flow cytometer (BD Biosciences).

Two batches of hMSCs from a 28 year old donor and a 24 year old donor were isolated separately. Human MSCs from the 28 year old donor were used in differentiation and periodic heat experiments, followed by a quantitative biochemical assay to analyze sulfated glycosaminoglycan (sGAG) and immunohistochemical (IHC) staining to obtain expressions of collagen I, II, X, aggrecan and heat shock protein 70. In order to investigate if results are donor specific or not, hMSCs of the 24 year old donor were used in another differentiation and periodic heat shock experiment, followed Western Blot to analyze expressions of collagen II, aggrecan and heat shock protein 70 (HSP70).

### Chondrogenic Differentiation in 3D Pellet Culture

To form a cell pellet, 2.5×10^5^ hMSCs in 0.5 ml medium were centrifuged down in a 15 ml conical tube at 150×g for 5 minutes at room temperature [41]. Chondrogenic differentiation was induced by chondrogenic medium composed of DMEM-high glucose, supplemented with 1% ITS+Premix (BD Bioscience), 1% penicillin-streptomycin, 100 μg/ml sodium pyruvate (Invitrogen), 50 μg/ml ascorbic acid-2-phosphate, 40 μg/ml L-proline, 0.1 μM dexamethasone, and 10 ng/ml recombinant human transforming growth factor-beta3 (TGF-β3) (Lonza, Walkersville, MD). Pellets were cultured at 37°C, 5% CO_2_ with medium changes every 2–3 days for 24 days.

### Heat Exposure

Human MSCs in chondrogenic(Chon) cultures were exposed to mild heat shock (HS) periodically on Day 2, 9, 16, and 23. The transient 1 hour heating at 41°C was performed using a cell culture incubator pre-calibrated with an accuracy of ±0.2°C as described in previous study [40]. The medium was changed after heating and the cells were back to the 37°C incubator. The control samples stayed in the 37°C incubator while the medium was changed at the same time as the heat shocked samples.

### Histology and Immunohistochemistry (IHC)

Chondrogenic pellets were harvested for histological analyses on Day 17 and 24 during differentiation. They were fixed using 10% acid-formalin with 70% ethanol [42] for 45 minutes at 4°C, and embedded in 2% agarose for easier handling, followed by dehydration in a graded ethanol series. The samples were then embedded in paraffin, and sectioned at a thickness of 5 μm using a Microm Rotary Microtome (Thermo Scientific, Walldorf, Germany). Thin sections were mounted on slides and immunohistochemical analyses of collagen type I, II, and X as well as chondroitin sulfate proteoglycan (CSPG) were performed to visualize collagen matrix distribution and confirm the aggrecan synthesis. In addition, the slides were stained for heat shock protein (HSP) 70 to observe the HSP expression induced with heat. Briefly, sections were deparaffinized, and then rehydrated through graded ethanol. For collagen staining, the samples were incubated in 0.5 mg/ml hyaluronidase for 30 minutes at 37°C. For HSP staining, antigen retrieval was performed in heated citrate buffer (10 mM sodium citrate, pH 6.0) at 95°C and the slides were allowed to cool to the room temperature. And then samples were incubated in 0.5 N acetic acid for 2 hours at 4°C. Endogenous peroxidase was quenched by 3% H_2_O_2_ in methanol for 10 minutes at room temperature. Nonspecific antibody binding was blocked by incubation with 10% normal horse serum (for COLI, II&X) or goat serum (for CSPG and HSP70) for 20 minutes at room temperature. Monoclonal mouse anti-human antibodies to collagen type I, collagen type II (IBEX Pharmaceutical Inc, Mont-Royal, QC, Canada), collagen type X (Developmental Studies Hybridoma Bank, University of Iowa, Iowa City, IA), CSPG, and HSP70 (Santa Cruz Biotechnology Inc, Santa Cruz, CA) were applied to sections at 4°C overnight, followed by biotinylated horse anti-mouse IgG for collagens or goat anti-mouse IgM for CSPG and HSP70 (Vector Laboratories, Burlingame, CA) for 30 minutes at room temperature. After antibody incubations, a peroxidase-based Vectastain ABC kit (Vector Laboratories) was used. Then samples were stained with diaminobenzidine (DAB) substrate kit (Vector Laboratories) and mounted with Clear-Mount aqueous mounting medium (Electron Microscopy Sciences, Hatfield, PA). Images were captured with a Zeiss Axiovert 25 C inverted microscope.

Semi-quantification of IHC staining images were performed using Image Pro Plus (Media Cybernetics). Color IHC images were converted to 8-bit gray scale images, followed by background subtraction and the average intensity measurement over all non-background regions. The intensity range is from 0 to 255 to represent the intensity of protein expression from low to high. Each IHC experiment included all culture conditions but only one sample per condition. After the semi-quantification analysis of IHC staining images, an intensity ratio of a chondrogenic&heating (Chon+HS) sample to a chondrogenic (Chon) specimen was calculated. IHC experiments and their semi- quantification image analysis were performed on three sets of biological samples. The average intensity ratios of Chon+HS to Chon for 5 proteins (i.e. Col I, II, X, aggrecan and HSP70) were reported as mean±SD.

### Assay for Glycosaminoglycans (GAGs)

Glycosaminoglycan (GAG) was initially detected by Safranin O staining. Though GAG synthesis was consistently higher in pellet samples treated with chondrogenic medium on Day 17 and 24 than samples in growth medium, Safranin O staining could not show a consistent trend of GAG synthesis by periodic heat shock in 3D pellet culture over three repeating experiments. Thus, a more quantitative sulfated GAG assay was applied to evaluate the effects of heat shock on GAG synthesis.

Pellet samples (n = 4) harvested on Day 10, 17 and 24 during chondrogenesis were weighed wet, lyophilized overnight, and then weighed dry. Samples were homogenized and digested in 13.22 mg/ml pepsin in 0.05 N acetic acid at 4°C for 48 hrs. The reaction was neutralized by 10× Tris buffered saline (pH 8). Total sulfated glycosaminoglycan (sGAG) content was then determined using quantitative 1,9-dimethylmethylene blue (DMMB) assay based on a chondroitin-6 sulfate standard curve. Absorbance was read at 595 nm using a BioTek Instruments microplate reader (Synergy 4, Winooski, VT). sGAG content was normalized to wet weight of each sample.

### Western Blot Analysis

Western blot (WB) analyses were performed using 3D pellet samples at day 17 and day 24 of differentiation. Briefly, cells were lysed in lysis buffer (150 mM NaCl, 1% NP-40, 0.5% Deoxycholic Acid, 0.1% SDS, 50 mM Tris pH 8.0) containing protease inhibitor cocktails (Sigma, St. Louis, MO). Proteins of lysate supernatant were separated with SDS-polyacrylamide gel electrophoresis, transferred to PVDF membrane (Bio-Rad, Hercules, CA) and immunoblotted with primary antibodies of collagen II, aggrecan, and HSP70, followed by incubation with the horseradish peroxidase (HRP) conjugated secondary antibodies. All antibodies were purchased from Santa Cruz Biotechnology (Santa Cruz, CA). Tetramethylbenzidine (TMB) substrate kit (Vector Laboratories, Burlingame, CA) was used to visualize the protein bands. Membranes were dried and scanned into digital images. Protein bands were analyzed quantitatively using Image Pro Plus software (Media Cybernetics). The protein expression levels were presented as ratios of total intensity of the protein bands normalized by that of the actin band from the same sample on the same membrane to minimize the protein loading variation.

### Statistical Analysis

All values were expressed as mean ± standard deviation (SD) and analyzed statistically using a two-tailed Student's t-test. The level of significance was set at p<0.05.

## Results

### Expression of Surface Markers in Isolated hMSCs

**Figure 1:**
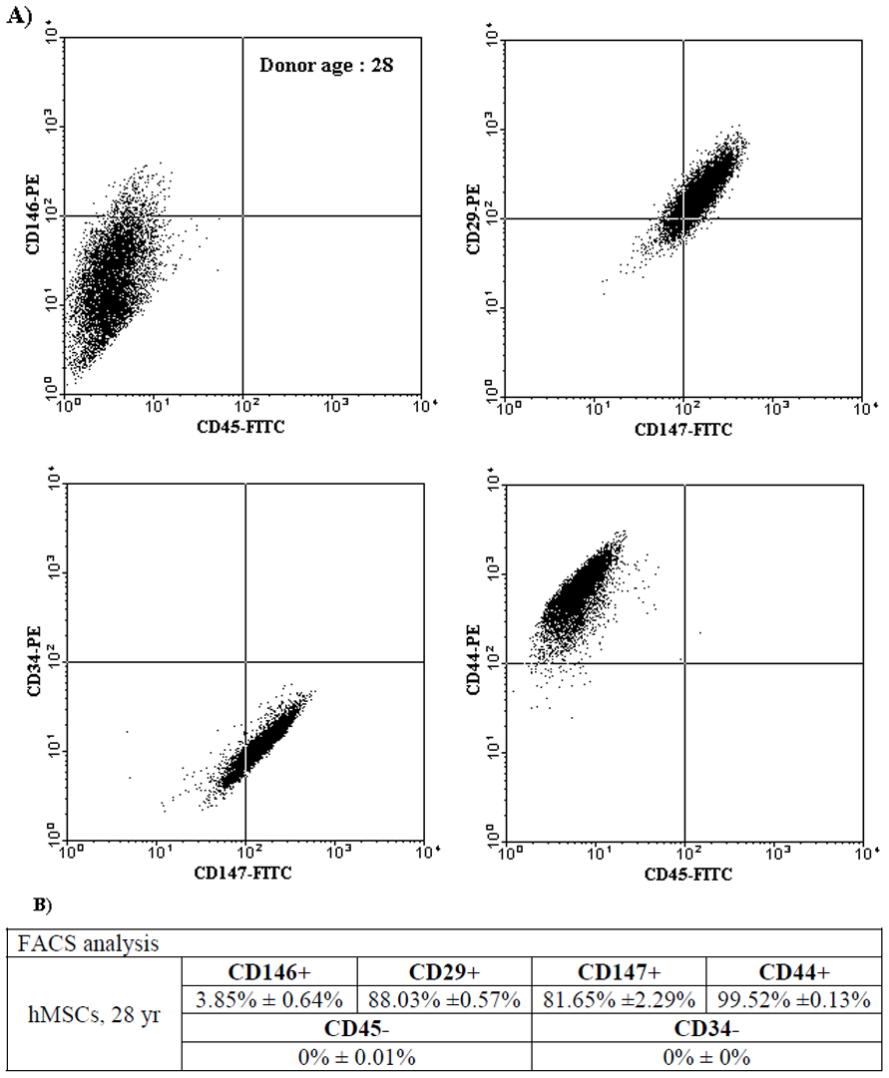
Characterization of hMSCs by flow cytometric analysis. (**A**) Isolated hMSCs were positive for surface markers CD146, CD29, CD147 and CD44, and negative for CD45 and CD34. (**B**) The individual percentages of each surface marker expressed in these cells from the quantitative FACS analysis. Data represent the mean ± SD (n = 3).

Passage 4 of isolated hMSCs was characterized by a set of surface markers including CD44 (hyaluronan receptor), CD29 (integrin β1), CD147 (extracellular matrix metalloproteinase inducer), CD146 (melanoma cell adhesion molecule), CD45 (leukocyte common antigen) and CD34 (lipopolysaccharide receptor). CD34 and CD45 are surface markers of the hematopoietic lineage, and CD146 is the surface marker of endothelial cell lineage, an epitope suggested as a biomarker for MSCs. Flow cytometric analysis showed that isolated hMSCs were slightly positive for surface marker CD146, positive for CD29, CD147 and CD44, and negative for CD45 and CD34 (**Fig. 1(A)**). Quantitative results from FACS analyses were shown in **Fig. 1(B)**. The individual percentages for surface markers CD146, CD29, CD147, CD44 expressed in the isolated hMSCs were 3.85±0.64%, 88.03±0.57%, 81.65±2.29%, and 99.52±0.13%, respectively, and none of cells were revealed to express CD45 and CD34 (0%).

### Accumulation of sGAG Content

**Figure 2:**
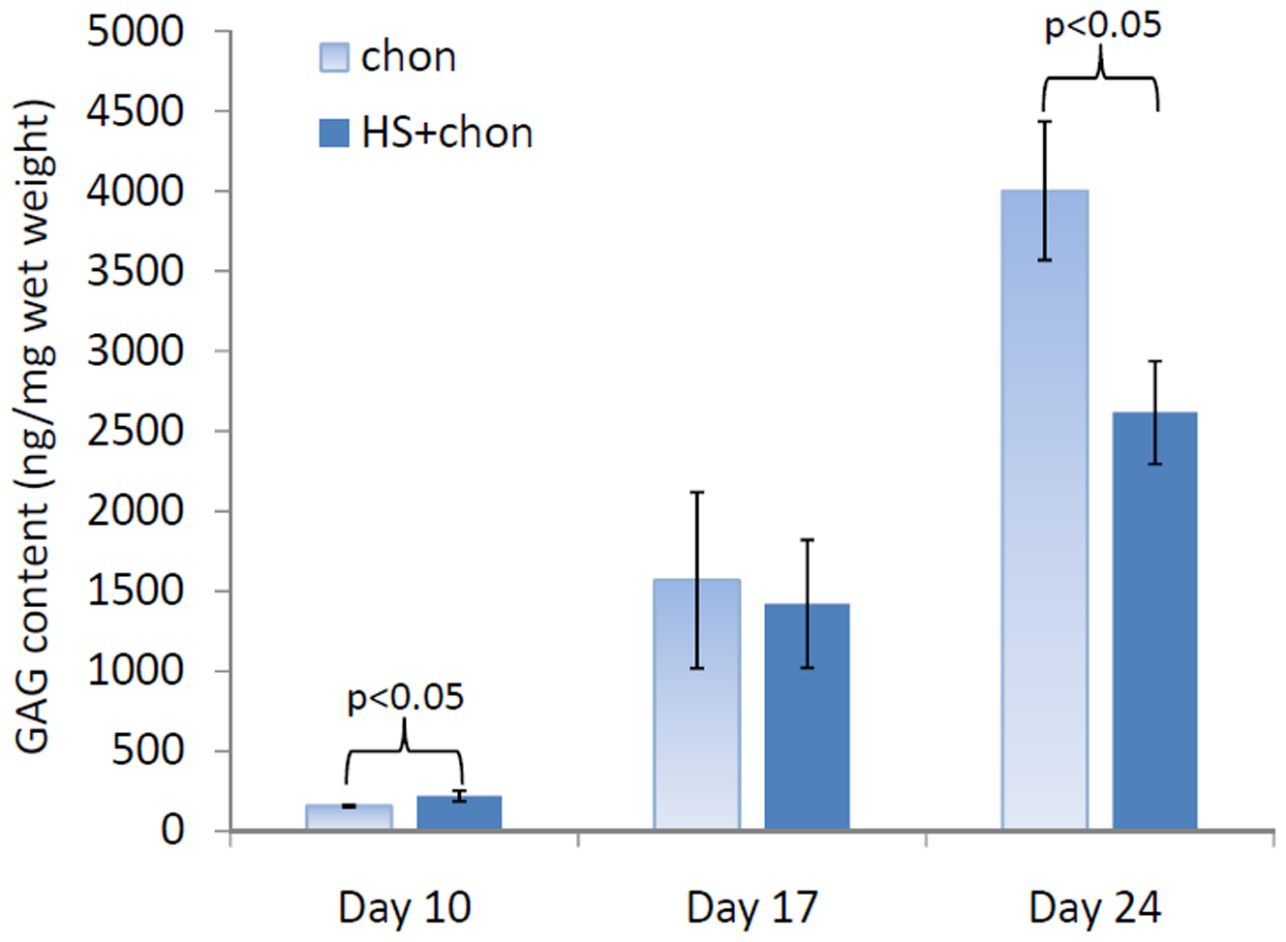
Sulfated GAG (sGAG) content normalized to wet weight in 3D pellet culture on Day 10, 17 and 24, with and without HS (chon: chondrogenic differentiated hMSCs, and chon+HS: chondrogenic differentiated hMSCs with heat shock). Data represent the mean ± SD (n = 4). P values were calculated using student t-test.

Figure 2 shows that periodic heat shock stimulates an approximate 1.5-fold increase in sulfated GAG (sGAG) content in 3D pellet culture on Day 10 relative to non heat-shocked controls. In addition, sGAG content builds up with time in chondrogenic culture in pellets through Day 24. Interestingly, Figure 2 also shows a significantly lower (∼1.5-fold) sGAG content in heat-shocked pellets than that in non heat-shocked pellets on Day 24 (**Fig. 2**).

### Distribution of Collagen Type II

**Figure 3:**
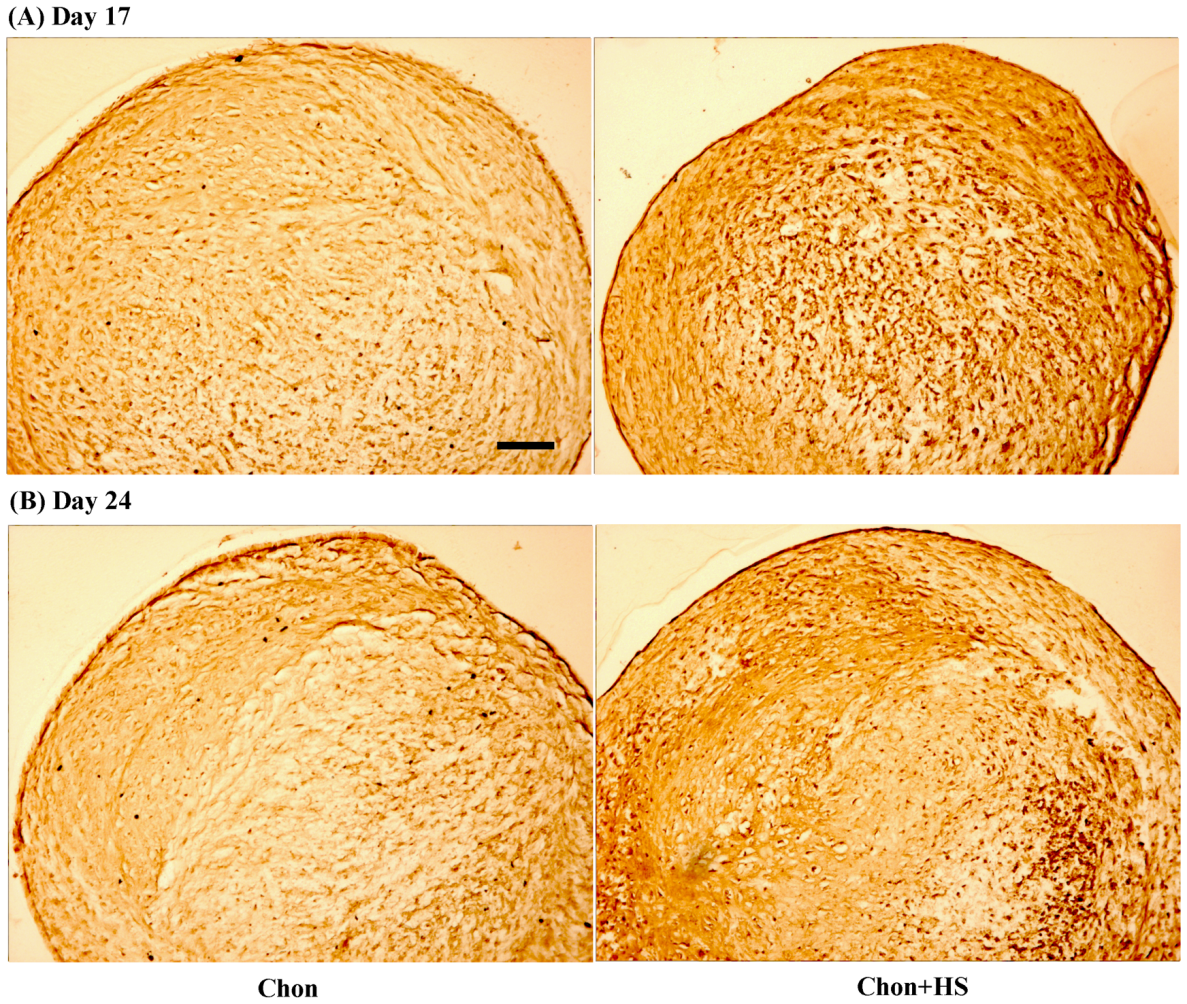
Representative images of immunohistochemical staining of collagen type II in pellet culture samples on (A) Day 17 (B) Day 24. Scale bar: 50 μm. (chon: chondrogenic differentiated hMSCs, and chon+HS: chondrogenic differentiated hMSCs with heat shock).

Immunohistochemical (IHC) analyses revealed an intense and relatively uniform positive staining of collagen type II throughout the whole pellet in chondrogenic pellets (**Fig. 3**). Meanwhile, increased staining of collagen type II in heat-shocked pellets than non heat-shocked pellets was observed on both Day 17 and 24 (**Fig. 3**), but the difference was less dramatic between samples with and without heat shock on Day 24.

### Aggrecan Synthesis

**Figure 4:**
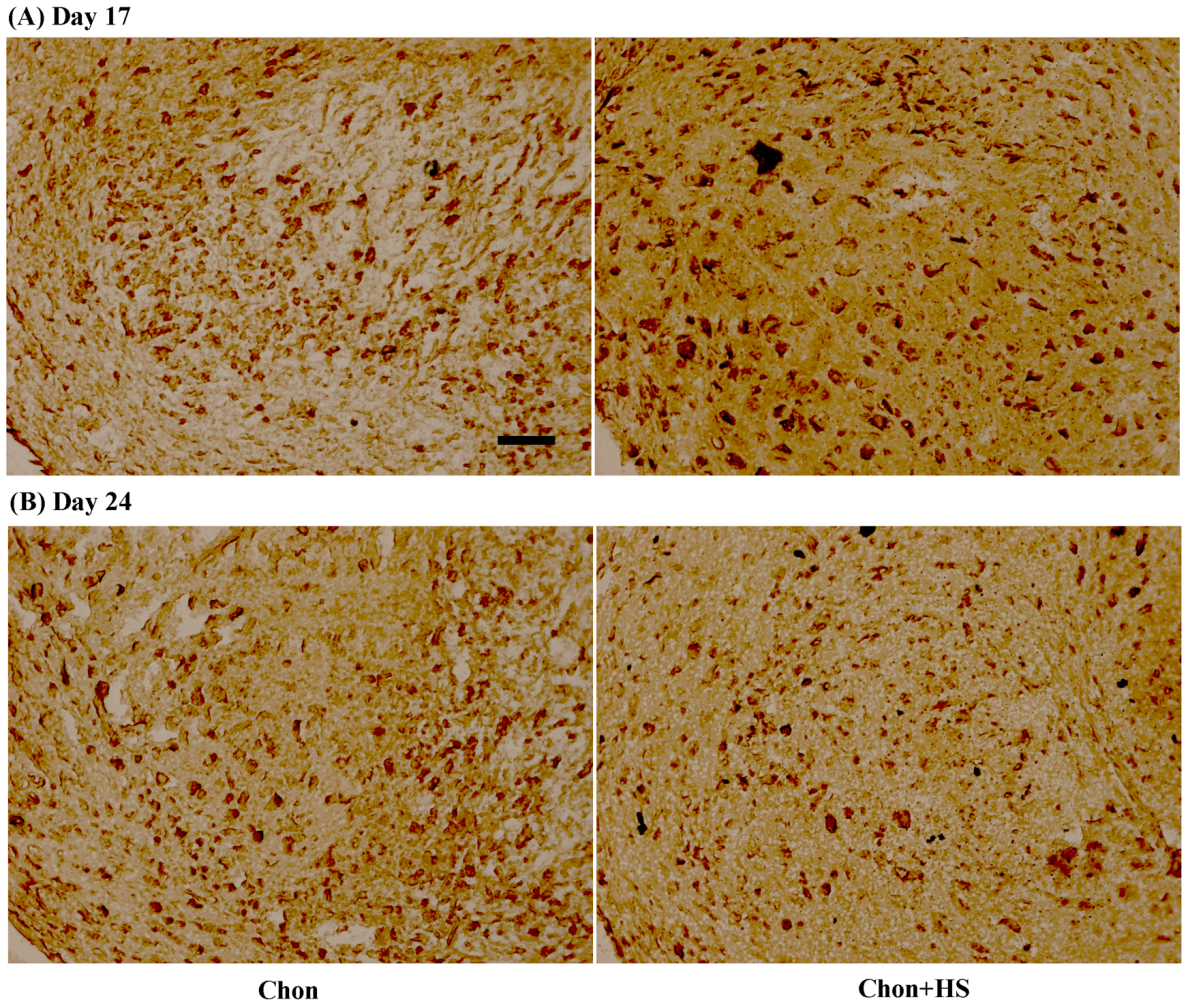
Representative images of immunohistochemical staining of aggrecan in pellet culture samples on (A) Day 17 (B) Day 24. Scale bar: 25 μm. (chon: chondrogenic differentiated hMSCs, and chon+HS: chondrogenic differentiated hMSCs with heat shock).

A modestly more intense staining of chondroitin sulfate proteoglycan (CSPG) by periodic heat shock on Day 17 in chondrogenic samples was observed. The staining for aggrecan was uniformly distributed and densely localized in the cell-associated regions (**Fig. 4**).

### Distribution of Collagen Type I

**Figure 5:**
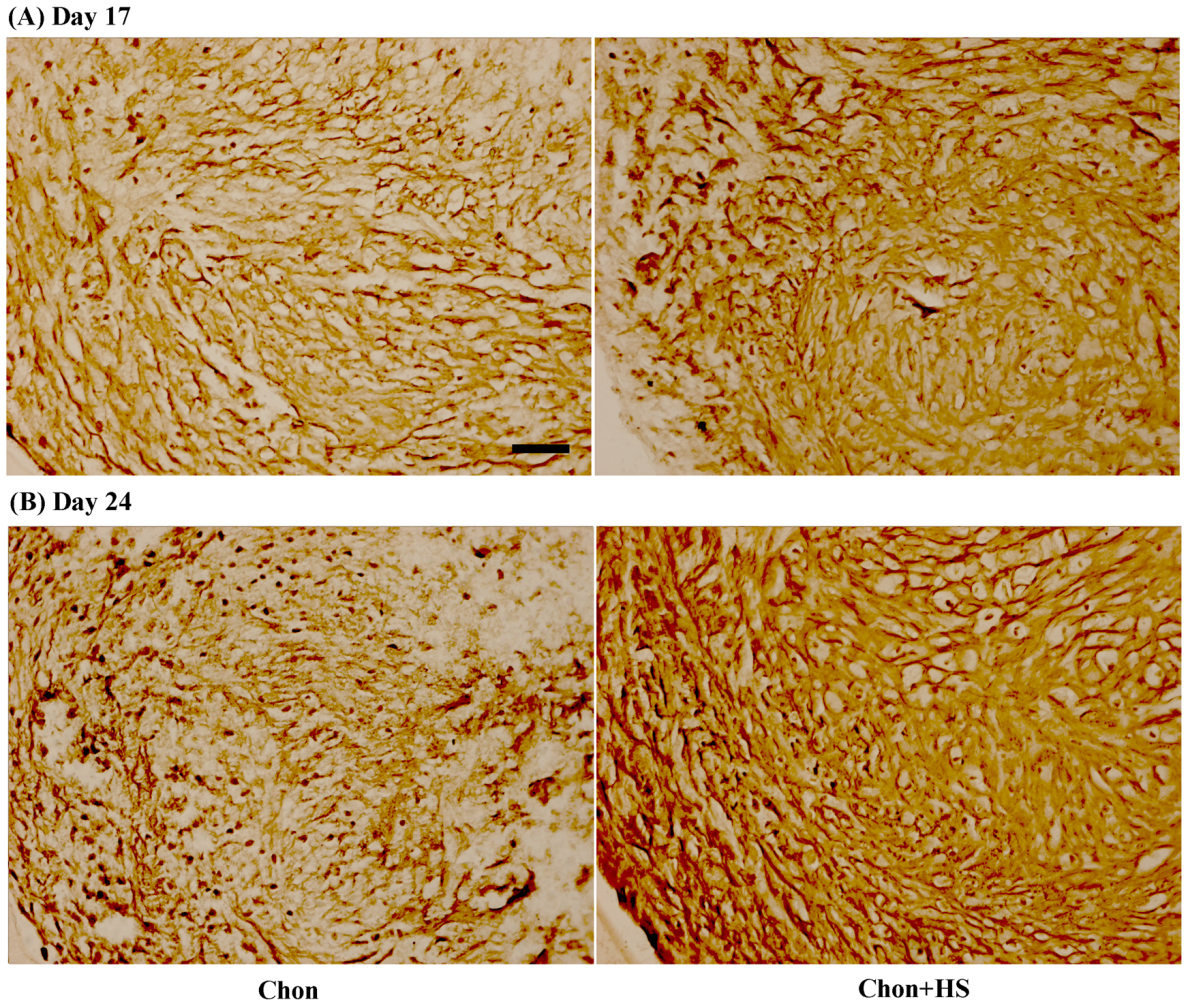
Representative images of immunohistochemical staining of collagen type I in pellet culture samples on (A) Day 17 (B) Day 24. Scale bar: 25 μm. (chon: chondrogenic differentiated hMSCs, and chon+HS: chondrogenic differentiated hMSCs with heat shock).

For collagen type I (Col I), positive staining was observed primarily around the pericellular regions (**Fig. 5**). Collagen type I expression was increased in heat shocked pellets than non heat-shocked ones on both Day 17 and 24 (**Fig. 5**).

### Distribution of Collagen Type X

**Figure 6:**
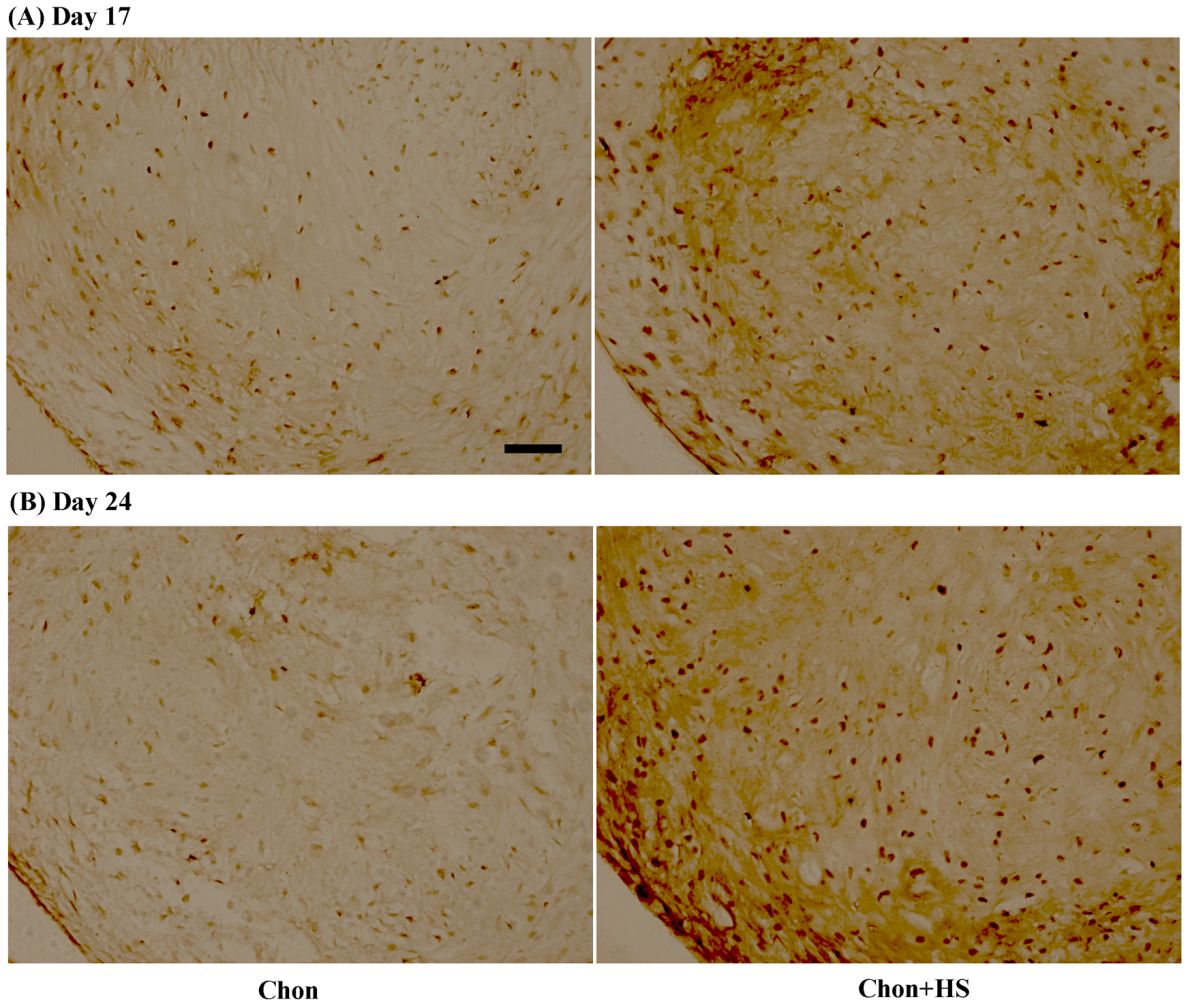
Representative images of immunohistochemical staining of collagen type X in pellet culture samples on (A) Day 17 (B) Day 24. Scale bar: 25 μm. (chon: chondrogenic differentiated hMSCs, and chon+HS: chondrogenic differentiated hMSCs with heat shock).

Low level and speckled staining of type X collagen (Col X) was observed throughout the chondrogenic pellets on both Day 17 and 24 (**Fig. 6**). Similarly, heat shock increased the expression of collagen type X in chondrogenic pellets on both Day 17 and 24, and the highest expression of Col X was observed in heat shocked chondrogenic samples of Day 24 (**Fig. 6**).

### Expression of Heat Shock Protein (HSP) 70

**Figure 7:**
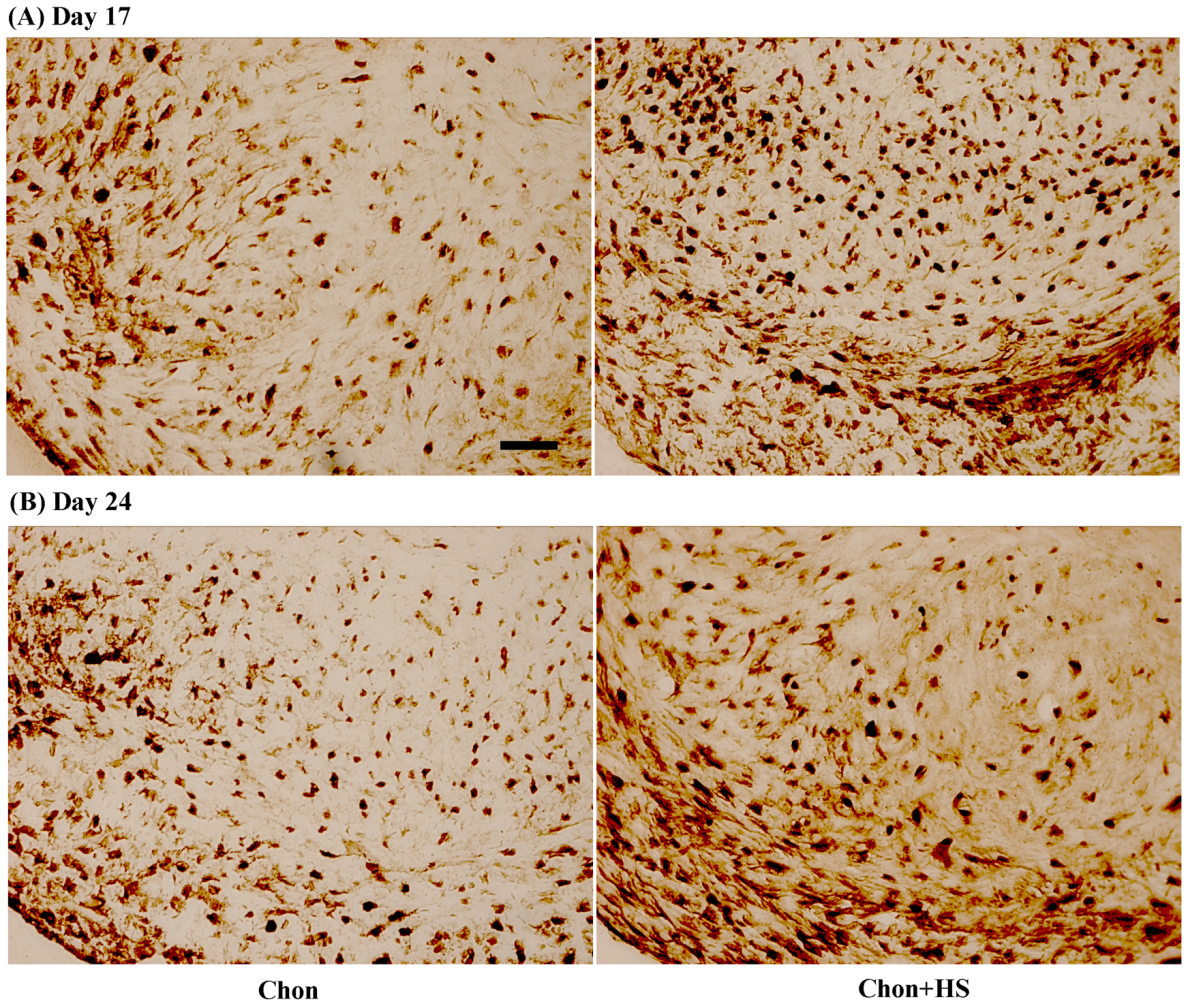
Representative images of immunohistochemical staining of inducible heat shock protein 70 (HSP70) in pellet culture samples on (A) Day 17 (B) Day 24. Scale bar: 25 μm. (chon: chondrogenic differentiated hMSCs, and chon+HS: chondrogenic differentiated hMSCs with heat shock).

Heat shock significantly increased the inducible HSP70 expression on both Day 17 and 24 in pellets cultured in chondrogenic medium, with more expression localized at the peripheral regions of the pellets (**Fig. 7**). The effect of heat shock was also more significant on Day 24 than that on Day 17 (**Fig. 7**).

### Semi-quantified intensity of Immunohistochemical (IHC) Staining

**Table 1** shows the semi-quantified results of the expression intensity of collagen type II, I, and X as well as aggrecan and HSP70 corresponding to their IHC staining images in Figures 3 to 7. The average ratio of the protein expression intensity at the Chon+HS (chondrogenic plus heat shock) culture condition to the Chon only over three sets of biological samples was also displayed in Table 1 as mean±SD. The semi-quantified IHC data shows that the periodic heat shock increases the expression of these five proteins about 20 to 40% during chondrogenic differentiation in 3D pellet cultures at Day 17 and 24 except the aggrecan expression on Day 24. Another common trend is that the effect of heating on the enhanced protein expression is slightly higher on Day 17 than that of Day 24 except the case of HSP70.

**Table 1:** Semi-quantified Immunohistochemical staining Intensity.

	Staining Intensity (Day 17)				Staining Intensity (Day 24)			
	IHC Staining Figures	Chon	Chon+HS	Average Ratio (Chon+HS/Chon)	IHC Staining Figures	Chon	Chon+HS	Average Ratio (Chon+HS/Chon)
Col II	3A	54	73	1.39±0.04	3B	54	71	1.36±0.06
Aggrecan	4A	127	143	1.29±0.10	4B	133	131	1.02±0.05
Col I	5A	99	121	1.33±0.14	5B	111	135	1.31±0.14
Col X	6A	113	138	1.28±0.08	6B	119	141	1.25±0.08
HSP70	7A	92	106	1.24±0.11	7B	87	111	1.31±0.05

Chon: Chondrogenic; HS: Heat Shock; Col: Collagen; n = 3 for average ratio.

### Expression of Chondrogenic Markers in hMSCs from the 24 Year Old Donor

**Figure 8:**
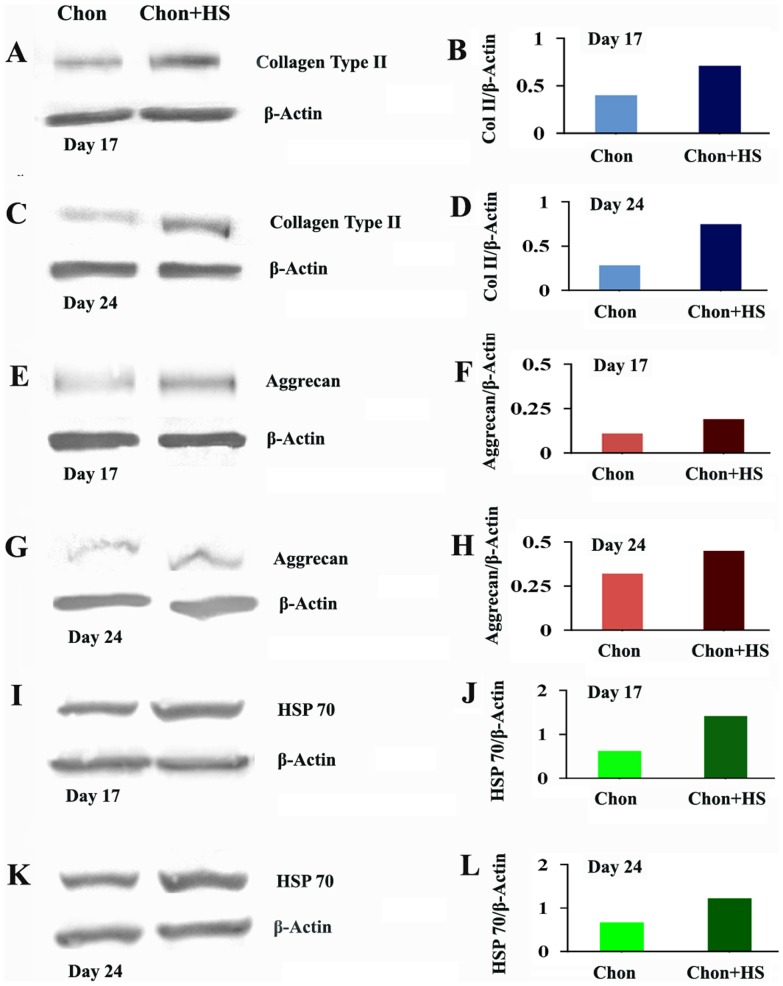
Western Blot analysis of collagen type II, aggrecan, and HSP70 expression in 3D chondrogenic pellet cultures using hMSCs from the 24 year old donor at Day 17 and Day 24. (A), (C), (E), (G), (I) and (K) are the images of Western blot membranes while (B), (D), (F), (H), (J) and (L) are their semi-quantified band intensities respectively (Chon: chondrogenic differentiated hMSCs, and chon+HS: chondrogenic differentiated hMSCs with heat shock).

To confirm that these findings are replicable among different donors, the exactly same chondrogenic experiments with the mild periodic heating were performed using hMSCs isolated from the 24-year-old donor. We then examined the expressions of collagen type II, aggrecan, and HSP70 in 3D chondrogenic pellets at day 17 and day 24 by Western blot analysis. As shown in **Fig. 8**, heat shock increased the expression of collagen type II, aggrecan, and HSP70 at Day 17 and Day 24. Among these three proteins, the enhancement effect of heating is the least on aggrecan. These results are consistent with the data obtained using hMSCs from the 28 year old donor by immunohistochemical analysis.

## Conclusions and Discussion

This study is the first to investigate the direct heat shock (HS) effects on chondrogenic differentiation of hMSCs in the pellet culture. It was shown that in-house isolated hMSCs that undergo chondrogenesis produced cartilage-like matrix rich in GAG, type II collagen and aggrecan. Periodic HS at 41°C for 1 hr once per week, was able to increase the sulfated GAG content at Day 10 (**Fig. 2**), as well as enhance the type II collagen production (**Fig. 3**) and aggrecan synthesis (**Fig. 4**) in 3D pellet culture at Day 17 of chondrogenic differentiation. In addition, these results are not dependent on specific stem cell donors. Our results support the hypothesis that mild HS may facilitate the earlier differentiation of hMSCs into chondrocytes, and thus it could be a simple and non-invasive approach for accelerating the regeneration of articular cartilage using stem cells.

MSCs have been shown to differentiate into chondrogenic lineage *in vitro* using a high cell density pellet culture system [43], which mimics the mesenchymal condensation in embryonic development of cartilage tissue, with transforming growth factor β (TGF-β) superfamily members, TGF-β1 or TGF-β3 [41], [44]. Typical protein or gene markers used for the chondrocyte phenotype identification include type II collagen and aggrecan [3], which are the two major extracellular matrix (ECM) components of cartilage. The effects of periodic HS on hMSC chondrogenesis in conventional 3D pellet culture were evaluated through ECM accumulation and compared between samples treated with or without HS.

The production of a cartilaginous matrix in the chondrogenic pellets after 2 weeks in culture was first verified by histological staining with Safranin O for sulfated GAG (sGAG) (data not shown). However, there were some variations within the group of chondrogenic pellet samples with or without effects of HS. Therefore, a quantitative sGAG assay was used to better evaluate the differences of sGAG production between heat shocked and non-heat-shocked pellet samples. Biochemical analyses revealed that periodic HS significantly increased sGAG content at early stage of differentiation in 3D pellet culture on Day 10 (**Fig. 2**). Consistent with earlier reports on chondrogenesis of hMSCs in pellet culture [41], abundant accumulation of sGAGs was found after 2 weeks and increased over time to 3-4 weeks in pellet culture. Interestingly, HS decreased the sGAG content in pellets on Day 24 (**Fig. 2**), which was possibly because that HS speeded up the chondrogenic differentiation of hMSCs in pellet culture and enhanced the earlier maturation which resulted in hypertrophic chondrocytes with less sGAG at late days. Another possible reason may be that multiple cycles of periodic heating enhanced the sGAG solubility in the surrounding medium. This possibility needs to be investigated further.

Immunohistochemical (IHC) staining was used to investigate HS effects on the protein expression of other ECM molecules present in chondrogenic pellet cultures of hMSCs, including type II collagen, type I collagen, and aggrecan. As shown by IHC analyses, periodic HS at 41°C significantly enhanced type II collagen expression on Day 17 and the effects were less significant on Day 24 (**Fig. 3**). Meanwhile, chondroitin sulfate proteoglycan (CSPG) staining was more intense in heat shocked pellets than non heat shocked ones on Day 17 (**Fig. 4**), and CSPG is a major component of aggrecan. Aggrecan was known as the most abundant proteoglycan in cartilage. No visible difference in the staining of aggrecan was detected at Day 24 between heat shocked and non heat shocked pellets. The semi-quantified IHC staining data in Table 1 support these findings quantitatively. The less increase of type II collagen or no increase in aggrecan expression by HS on Day 24 of chondrogenic differentiation may be another indication of fast maturation driven by HS into the stage of hypertrophic chondrocytes. It could also be the result from their increased solubility in the medium due to the periodic heating. Thus the results from sGAG, type II collagen and aggrecan synthesis altogether support our hypothesis that HS may accelerate the differentiation of hMSCs to chondrocytes in pellet culture.

Furthermore, since type I collagen was regarded as an important ECM molecule during early chondrogenesis [45] and a marker for fibrocartilage [46], we also assessed its expression in this study. Type I collagen appeared to be unchanged during chondrogenic differentiation with a substantially lower level than either type II collagen or aggrecan when Figure 5 was compared with Figure 3 and 4. Gene expression study also revealed that type I collagen was present throughout the differentiation in hMSC chondrogenic pellet culture [47]. Interestingly, HS was able to increase the expression of collagen type I (Col I) on both Day 17 and 24, and more Col I was seen on Day 24. It suggests that a fibrocartilage-like phenotype that usually present in chondrogenic MSC pellet cultures as reported in previous studies [41], [45] may be further enhanced by heat shock on Day 24. The underlying mechanisms are unclear, but possibly the continued synthesis process of collagen type I was initiated by the temperature increase on differentiated hMSCs. The co-expression domains in the staining patterns of type I and II collagens may indicate that cells were experiencing a transition phase between fibroblastic and chondrocytic phenotypes.

In order to confirm our speculations that HS may accelerate the maturation process and lead to a hypertrophic chondrocyte stage in a much shorter differentiation period, the expression of type X collagen was evaluated. Type X collagen is considered as a marker for late-stage chondrocyte hypertrophy [1], [48] that is associated with endochondral ossification during skeletal development, in which mature chondrocytes undergo a terminal differentiation process of hypertrophy, mineralization, and apoptosis, and eventually the cartilage is replaced with mineralized bone [45], [49]. Figure 6 shows that HS significantly increased type X collagen expression on both Day 17 and 24, as visualized by more brown dots in heat shocked pellets. HS caused a faster progression of some hMSCs into the hypertrophic stage as early as Day 17 especially at the peripheral regions of the pellets (**Fig. 6**). This supports our observation of the decrease in sGAG synthesis at Day 24 by HS (**Fig. 2**). Noticeably, only some of the cells reached a sufficient chondrogenic differentiation state faster and proceeded toward hypertrophy, which may be due to the nonhomogeneous hMSC population with varying differentiation potentials.

On the other hand, 41°C may not be the optimized heating temperature for enhancing the chondrogenesis although it proved to be most effective for osteogenesis in the previous study [40]. The goal in articular cartilage tissue engineering for OA patients is to generate hyaline cartilage, thus searching ways to diminish the formation of hypertrophic chondrocytes in MSC chondrogenesis would be very necessary [41], [50]. Co-culture of chondrocytes with MSCs in pellet culture, encapsulation of MSCs in tailored hydrogel, or chondrogenesis of MSCs in hypoxia condition have all been shown to decrease the hypertrophic markers (e.g. collagen type X, etc.) [51]. Transferring a basic fibroblast growth factor (FGF-2) gene to MSCs reduced both type I and X collagens *in vitro* and was beneficial for the maintenance of the differentiation of MSCs in a prehypertrophic state [52]. Using lower temperature (e.g. 39°C, body temperature during exercise [53]) or less frequent heat shock may be more relevant and will be further studied to reach an optimum turnout of differentiation.

To further study the mechanisms of HS-enhanced hMSC chondrogenic differentiation, we also evaluated the expression of heat shock protein 70 (HSP70). HSP expression after heat exposure has been studied intensively in all cell processes including differentiation. HSPs work to help correct misfolded proteins under stresses or fold newly synthesized proteins during normal development. HSP70 (70 kDa) is one of the most studied and consistently induced HSPs in mammalian cells. In addition, HSP70 expression was found to be closely related with chondrocyte activities including the inhibition of apoptosis [37], [39]. As shown in Figure 7, in non-heat-shocked differentiated hMSC pellets, inducible HSP70 was present at a low level. Inducible HSP70 expression was significantly enhanced by HS at both Day 17 and 24, with more significance observed on Day 24 (**Fig. 7**), although there was no observable difference in total HSP70 (constitutive HSC70 and inducible HSP70) expression probed by another antibody (Santa Cruz Biotechnology Inc, sc-24) between heated and non-heated pellets on Day 17 and 24 (data not shown). Inducible HSP70 over-expression may be partially responsible for the HS-induced acceleration of chondrogenic differentiation. More studies using HSP70 siRNA/shRNA need to be carried out to investigate the role of HSP70 in this process. It was also observed that inducible HSP70 expression was localized more to the peripheral regions of the pellets, which coincided with collagen type X expression in heat shocked pellets (**Figs. 6** **&** **7**). We speculate that hMSCs in the hypertrophic chondrocyte stage or at late days of differentiation might be under more cellular stresses. Therefore, more HSP70 was induced to prevent further chondrocyte hypertrophy which leads to apoptosis later on. The relationship between inducible HSP70 over-expression and collagen type X synthesis needs to be further investigated.

In summary, the overall results of this study showed that periodic HS accelerated chondrogenic differentiation of hMSCs. We had previously reported the promotion of osteogenesis in hMSCs by periodic HS [40]. Taken together, these two reports show that HS may be useful for repairing full-thickness joint defects involving both cartilage and bone. For example, in the late stage of OA, the regeneration of cartilage, hypertrophic cartilage and bone is required from differentiation of implanted hMSCs. The intensity, interval and duration of heat stimulation used in this study can be further optimized to have well-controlled effects and achieve the desired differentiation results using hMSCs. Further in vitro and in vivo experiments are needed to figure out how to manipulate the maturation of hMSC differentiation by different thermal doses. On the other hand this study may provide a scientific base on how to use thermal treatments to facilitate the regeneration of fibrocartilage (e.g. intervertebral discs).
